# 30-Month Pot Experiment: Biochar Alters Soil Potassium Forms, Soil Properties and Soil Fungal Diversity and Composition in Acidic Soil of Southern China

**DOI:** 10.3390/plants11243442

**Published:** 2022-12-09

**Authors:** Hao Xia, Bo Liu, Muhammad Riaz, Yuxuan Li, Xiangling Wang, Jiyuan Wang, Cuncang Jiang

**Affiliations:** 1 Microelement Research Center, College of Resources and Environment, Huazhong Agricultural University, Wuhan 430070, China; 2 Key Laboratory of Fertilization from Agricultural Wastes, Ministry of Agriculture and Rural Affairs, Institute of Plant Protection and Soil Fertilizer, Hubei Academy of Agricultural Sciences, Wuhan 430064, China; 3 College of Resources and Environment, Zhongkai University of Agriculture and Engineering, Guangzhou 510225, China; 4 The Key Laboratory of Oasis Ecoagriculture, Xinjiang Production and Construction Corps, Shihezi University, Shihezi 832000, China

**Keywords:** biochar treatment, soil properties, fungal community, potassium levels

## Abstract

Biochar has a significant impact on improving soil, nutrient supply, and soil microbial amounts. However, the impacts of biochar on soil fungi and the soil environment after 30 months of cultivation experiments are rarely reported. We studied the potential role of peanut shell biochar (0% and 2%) in the soil properties and the soil fungal communities after 30 months of biochar application under different soil potassium (K) levels (100%, 80%, 60%, 0% K fertilizer). We found that biochar had a promoting effect on soil K after 30 months of its application, such as the available K, water-soluble K, exchangeable K, and non-exchangeable K; and increments were 125.78%, 124.39%, 126.01%, and 26.63% under biochar and K fertilizer treatment, respectively, compared to control treatment. Our data revealed that *p_Ascomycota* and *p_Basidiomycota* were the dominant populations in the soil, and their sub-levels showed different relationships with the soil properties. The relationships between *c_sordariomycetes* and its sub-level taxa with soil properties showed a significant positive correlation. However, *c_Dothideomycetes* and its sub-group demonstrated a negative correlation with soil properties. Moreover, soil enzyme activity, especially related to the soil C cycle, was the most significant indicator that affected the community and structure of fungi through structural equation modeling (SEM) and redundancy analysis (RDA). This work emphasized that biochar plays an important role in improving soil quality, controlling soil nutrients, and regulating fungal diversity and community composition after 30 months of biochar application.

## 1. Introduction

The citrus industry is the world’s largest fruit industry and occupies a very important position in global trade. China is one of the foremost producers of citrus. The citrus production area and fruit output of China surpassed Brazil’s, making China the world’s largest citrus producer [1]. Compared with of citrus-producing countries, there is still a big gap in cultivation methods, planting management level, and variety [2]. Citrus is mainly cultivated in the southern tropical region of China. This area is dominated by acidic soil, and the plants are prone to disease. In addition, acidic soil often causes aluminum toxicity and manganese toxicity, which can inhibit the absorption of plant nutrients, hinder root growth, and inhibit the growth of citrus seedlings [3,4].

Potassium (K) is a macronutrient and stress resistance element that can improve the quality of citrus fruits and their ability to adapt to the external environment [5,6]. Unreasonable fertilization measures can also reduce fruit quality, the amount of juice, sour taste, and lower solids [7]. The majority of K in the soil is structural K that exists in the mineral crystal lattice, which is difficult for plants to use directly, and only a small amount of water-soluble K and exchangeable K can be directly absorbed and utilized by plants [8,9]. In general, K deficiency is very common in the soil of China, and K fertilizer is a non-renewable resource, and potash resources are also very scarce in China [10]. In addition, the high temperature and rain in the southern region and the strong soil leaching often cause soil K deficiency [11]. Therefore, we urgently need to find a new material that can replace some of the K fertilizer used or can improve the availability of soil K.

In recent years, as an emerging material, biochar, has been used in agriculture, environmental conservation, energy, and other fields. As a solid material, biochar is obtained by the thermochemical conversion of biomass in an oxygen-limited environment [12,13]. Biochar also contains available nitrogen (N), phosphorus (P), K, and a variety of other inorganic salt ions, and so can be used as fertilizer [14,15,16]. Due to its characteristics and the ability to supply K, biochar not only directly affects the properties of the soil after being applied to the soil, but also indirectly affects the soil’s properties through changes to the soil environment (temperature, moisture, and pH value). The total K content in biochar is 4–91 g·kg^−1^. It can directly supply available K to the soil, break the balance of different forms of K in the soil, and so affect the forms of K present [17,18]. The water-soluble K in biochar accounts for 3.5–100% of the total K, which significantly increases the content of soil available K and promotes plant growth [19,20]. In addition, biochar affects the forms of K in the soil by changing the physical and chemical properties of the soil (temperature, moisture, pH, and soil cation exchange capacity) [21,22]. Soil enzymes are closely related to the metabolic activity of microorganisms and the biogeochemical cycle of nutrients [23,24]. A large number of studies have found that biochar can significantly increase dehydrogenase activity [25,26]. Soil microorganisms are an important part of the soil ecosystem and are closely related to the biogeochemical cycle of the soil, such as the formation of soil aggregates, and the fixation and release of nutrients. The microbial community and its abundance in the soil are the key factors for soil nutrient cycling, and biochar provides a suitable habitat for beneficial soil microorganisms [27,28,29]. Wang et al. [30] found that biochar improves the activity of some extracellular enzymes in the soil carbon cycle and sulfur cycle. Lin et al. [31] and Zhang et al. [32] also found that the application of peanut shell biochar increases the soil nutrients and subsequently can increase crop yield.

Fungi are important members of soil microorganisms and play an important role in the energy flow and material cycle of the soil ecosystem [10]. It has been well documented that *Penicillium* fungi can decompose cellulose, lignin, and starch in soil [33]. Biochar is rich in organic carbon, which can stimulate the fungal community, promote soil nutrient cycling, and improve the activity of the soil fungal community [34]. Wang et al. [35] found that when the application rate of biochar was 0.5%, it was helpful to promote the metabolic activities of microorganisms. Luo et al. [36] showed that biochar significantly increased the relative abundance of soil fungi through field experiments. Hu et al. [37] found that the addition of biochar to red soil reduced fungal diversity in the short term.

Most of the relevant studies have explored the short-term effects of biochar on soil’s microbial diversity and its community structure, and few studies have explored the relationship between fungal diversity and the soil’s chemical properties under different biochar and K fertilizer treatments during long-term experiments. To achieve this, we used acidic soil as the research material to explore the changes in the forms of K in acidic soils during the over two years of peanut shell application, and the effect of biochar on soil fungi. Using dynamic sampling observation and ITS high-throughput sequencing technology, the experiment was carried out to evaluate the long-term reduction in fertilizer input and as basic research for the application of biochar. We hypothesized: Biochar continuously improves soil’s pH, K level, organic matter, and enzyme activity. Will biochar would have same effect on these parameters, especially related to the C cycle, after 30 months of application? This study involved the application of biochar to soil to check the relationship between soil chemical properties and soil fungi under different biochar and K fertilizer treatments and to further identify sensitive microbial taxa under biochar and K fertilizer treatments after 30 months of biochar application.

## 2. Results

### 2.1. The Changes in Soil Chemical Properties over Time under Biochar and Chemical Fertilizer Treatments

According to the results of Table 1, the chemical properties of soil are dynamic. They changed differently with the different treatments during 30 months of treatment. The addition of biochar significantly (*p* < 0.05) increased the pH, available nutrients, and SOM compared to without biochar treatment (Table 1). The pH was rapidly increased (*p* < 0.05) during the sixth month of treatment application. The application of biochar increased pH by 0.38–0.51 units, compared to fertilizer alone, and pH showed a decreasing trend with K fertilizer addition (Table 1). The biochar promotion effect was weaker over time: the soil pH increased by 0.09–0.20 units over the 30th months of the experiment (Table 1). A significant increase in SOM was observed in biochar–fertilizer treatment compared with fertilizer-only treatment. After 6 months, biochar increased the content of SOM (*p* < 0.05), and SOM increased by 67.92–140.36% under biochar–fertilizer treatment relative to no biochar treatment, from 15.07–17.27 to 29.00–35.06 g·kg^−1^. However, SOM only increased from 7.22–8.06 to 15.16–16.40 g·kg^−1^. It is worth noting that the effects of biochar only caused minor changes in AN and AP among biochar–fertilizer treatments.

Interestingly, soil pH and AP showed an apparent increasing trend during all cultivation stages. The pH and AP increased from 4.55–5.11 and 5.77–12.40 mg·kg^−1^ after 6 months to 6.53–6.87 and 26.12–35.94 mg·kg^−1^ in the 30th month (Table S3). However, the contents of SOM and AN showed opposite trend compared to that of soil pH during the incubation stage (Table 1). Compared with the contents of SOM and AN in the 6th month, the SOM and AN decreased 53.33–56.15% and 14.93–27.37% in the 30th month (*p* < 0.05) (Table S3). Moreover, there were remarkable effects of biochar, K fertilizer and time on pH, SOM, and AN during all cultivation processes (*p* < 0.05) (Table S5). In addition, the interaction effect of biochar and time had a more significant impact on pH, SOM, AP, and AN. Only SOM was affected by the Interaction effect of biochar, K fertilizer, and time (Table S5).

### 2.2. The Changes in K Forms under Biochar and Chemical Fertilizer Treatments

**Figure 1:**
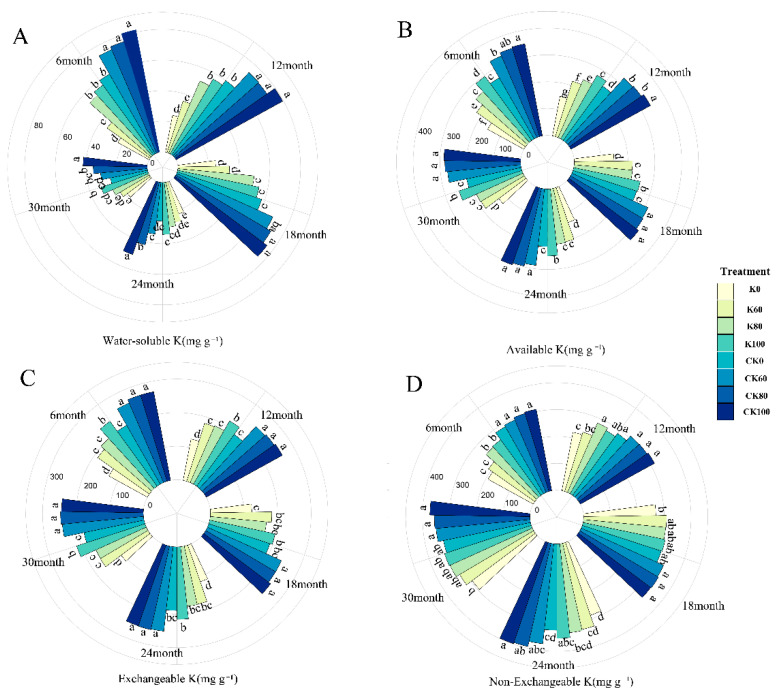
Water-soluble K (**A**), available K (**B**), exchangeable K (**C**), and non-exchangeable K (**D**) during the incubation under different biochar and K fertilizer treatments. (Sampling time: 6 months, 12 months, 18 months, 24 months, 30 months). Different lowercase letter indicate significant differences according to Duncan’s test (*p* < 0.05).

From the results of Figure 1, we observed that the biochar–fertilizer together had a better effect on different soil K forms compared with fertilizer or biochar alone (*p* < 0.05). The application of biochar and K fertilizer increased AK, WK, EK, and NEK by 126.14%, 244.32%, 105.09%, and 60.31% in AK, WK, EK, and NEK compared to without biochar and K fertilizer treatments after 6 months (Figure 1). The impacts of biochar and K fertilizer on soil K forms were observed after 30 months. There was a notable promotion of soil K forms in CK100 treatments relative to K0 treatment. The AK, WK, EK, and NEK increased by 125.78%, 124.39%, 126.01%, and 26.63% (Figure 1).

During the incubation stage, the AK, WK, and EK levels were lowered in different treatments (Figure 1 and Table S4). From 6 to 30 months, the AK, WK, and NEK significantly decreased by 18.00–18.13%, 19.85–47.76%, and 9.27–17.67%. Furthermore, the NEK showed an increasing trend during incubation and reached its maximum after 30 months (Figure 1 and Table S4). What is more, there were obvious main effects (biochar, K fertilizer and time factor) on AK, WK, EK, and NEK during all incubation time. The joint effect of biochar and K fertilizer had a more remarkable effect on the soil K component (Table S5). Only AK was affected by the combined effect of biochar, K fertilizer, and time (Table S5).

### 2.3. The Changes in Fungi over Time under Biochar and Chemical Fertilizer Treatment

#### 2.3.1. The Response of Alpha Diversity to Different Treatments

**Figure 2:**
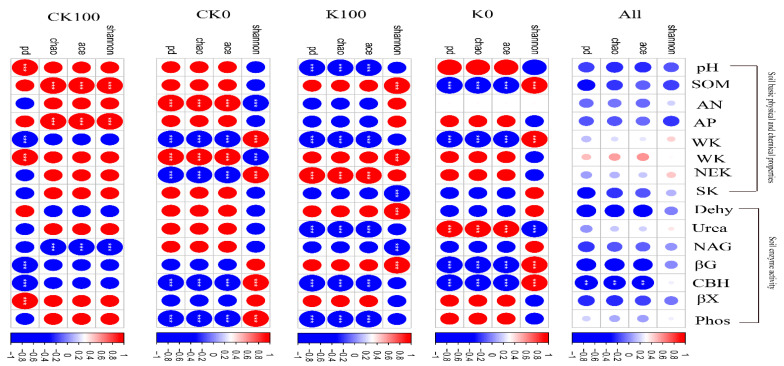
Panel shows heatmaps of Spearman’s rank correlation coefficients of fungal alpha-diversity metrics with soil physicochemical properties for the entire set of soil samples (All) and subsets of different soil depths (K0, K100, CK0, and CK100). *** Correlation is significant at the 0.001 level. ** Correlation is significant at the 0.01 level. Blue indicates a negative correlation, and red indicates a positive correlation.

The results of the pot experiment showed that Ace, Chao, and Pd were not significantly affected by biochar and K fertilizer addition (Figure S1). In general, microbial diversity was not significantly correlated with soil chemical properties (except for CBH) (Figure 2). However, a separate analysis of each treatment revealed an interesting result. There was a close association between soil physicochemical properties and fungal diversity under each treatment (Figure 2). In addition, this relationship changed with the addition of biochar and K (Figure 2). For example, the soil SOM was significantly negatively correlated with alpha diversity (Ace, Chao, and Pd) under K0 treatment (*p* < 0.05), and the soil SOM showed a positive correlation with alpha diversity (Ace, Chao and Pd) with biochar addition (Figure 2).

#### 2.3.2. The Associations between Individual Taxa of Fungi and Selected Soil Properties

**Figure 3:**
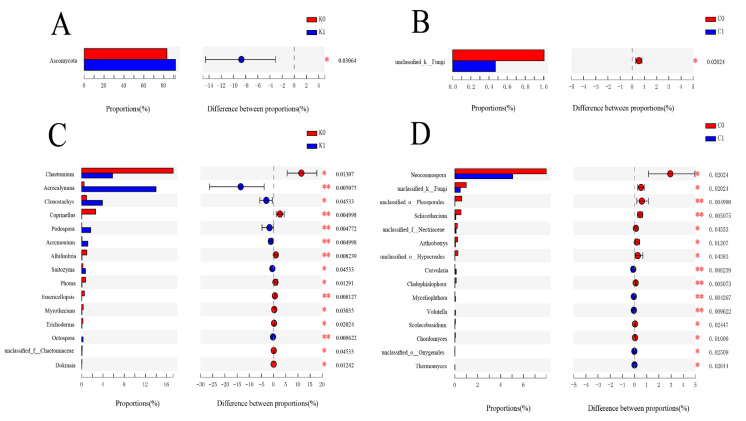
Two groups of comparison based on phylum and genus levels with K fertilizer and biochar addition. (**A**,**B**) and (**C**,**D**) represent the statistical results at phylum and genus levels, respectively. *p* < 0.05 marked as *, *p* < 0.01 marked as **.

All the dominant fungi phyla were detected in the soil samples. The abundant phyla were *p_Ascomycota*, followed by *p_Mortierellomycota* and *p_Basidiomycota* (Figure S2A). In addition, different fungal communities had different changes in response to biochar and K fertilizer treatments (Figure S2B). According to the two groups of comparisons based on the phylum and genus levels, we found that the relative abundance of *p_Ascomycota* was significantly increased on the phylum level with the addition of K fertilizer, and most fungi decreased in abundance with the addition of K fertilizer and biochar (Figure 3). Details of the two groups of comparisons are given. There were 9 and 10 fungal taxa increased on the genus level with K fertilizer treatment and biochar treatment in the top of 15 taxa, respectively (Figure 3).

**Figure 4:**
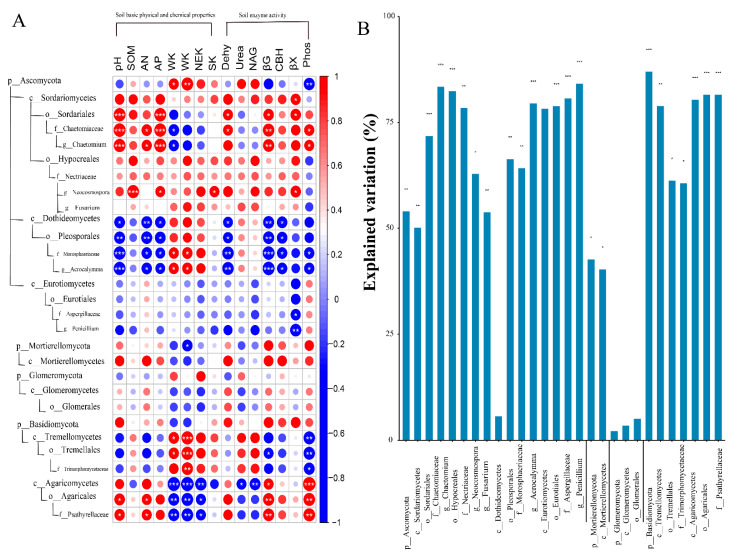
Panel (**A**) shows the heatmap of Spearman’s rank correlation coefficients between relative abundances of major fungal taxa from phyla to genera (dominance of the relative abundance on average) and soil properties. *** Correlation is significant at the 0.001 level. ** Correlation is significant at the 0.01 level. * Correlation is significant at the 0.05 level. Blue indicates negative correlation, and red indicates positive correlation. Panels (**B**) shows the stepwise multiple regression (SMR) showing the total explanation rate of environmental variables to the richness of each species. *** Correlation is significant at the 0.001 level. ** Correlation is significant at the 0.01 level. * Correlation is significant at the 0.05 level.

The pot experiment’s results regarding different levels of fungi taxa, *p_Ascomycota* and *p_Basidiomycota*, revealed significant correlations with one or more soil properties (Figure 4A). *p_Ascomycota* and its sub-level taxa (*c_Sordariomycetes* and *c_Dothideomycetes*) were mainly affected by soil properties. Generally, the relationships of *c_Sordariomycetes* and their subordinate taxa with soil properties (pH, AP, Dehy, and βG) showed a significantly positive correlation (Figure 4A). However, the *c_Dothideomycetes* and their subordinate taxa also had negative correlations with soil properties (pH, AN, AP, Dehy, βG, and CBH) to some extent. Unlike *c_Sordariomycetes* and its subgroups being negatively correlated with various soil K forms, the *c_Dothideomycetes* and its sub-classification groups were positively related to various soil K forms (Figure 4A). The fungi taxa of *p_Basidiomycota*, including *c_Agaricomycetes* and *c_Tremellomycetes* and their subordinate taxa, were mainly related to soil properties (different soil K forms, βG, and phosphatase). A negative effect of *c_tremellomycetes* and its sub-level taxa with phosphatase and a positive effect on different soil K forms was observed from the results of the pot experiment (Figure 4A). Furthermore, we used stepwise multiple regression (SMR) approaches to predict the importance of soil properties on the abundance of taxa of fungi at different levels (Figure 4B). The results of the pot experiment also confirmed that *p_Basidiomycota*, *p_Ascomycota*, *p_Mortierellomycota,* and their sub-level taxa were significantly influenced by the soil environment (Figure 4B). However, the taxa of *p_Glomeromycota* were not affected by soil properties.

**Figure 5:**
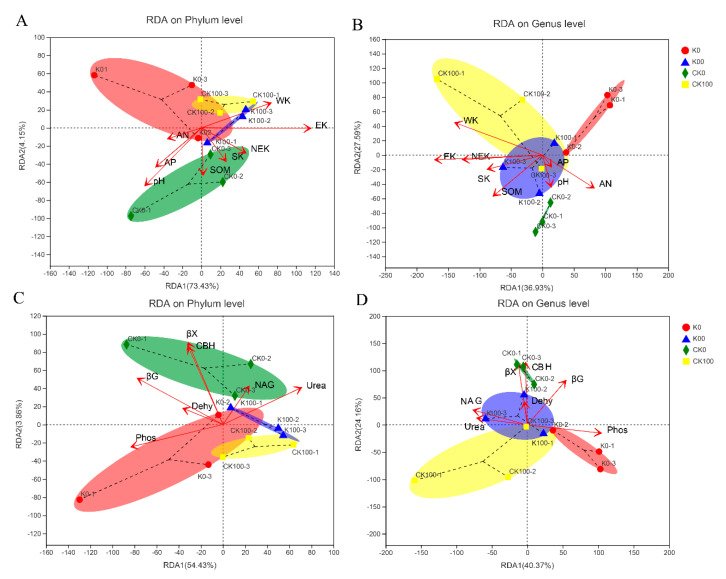
Results of redundancy analysis of acidic soils. (**A**,**B**) represent soil chemical properties on phylum and genus levels. (**C**,**D**) represent soil enzyme activity on phylum and genus levels.

**Figure 6:**
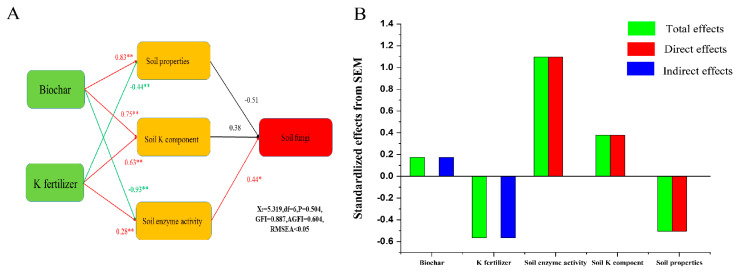
The structural equation modeling (SEM) shows the influences and effects of biochar and K fertilizer on soil properties, soil K components, soil enzyme activity, and soil fungi. The numbers above the arrows denote the standardized path coefficients. The red and yellow arrows indicate positive and negative effects, respectively (**A**). The standardized regression and influence effects of the structural equation modeling (SEM) (**B**). Stars denote significance at *p* < 0.05 and *p* < 0.01 probability levels (* and **, respectively).

According to RDA results, EK, βG, CBH, and βX had a clear influence on fungi in the soil microbial community at the phylum level (*p* < 0.05) (Figure 5 and Table S6). Moreover, the results indicated that WK, NEK, CBH, βX, and phosphatase each had an obvious influence on soil microbial communities at the genus level (*p* < 0.05) (Figure 5 and Table S6). Structural equation modeling (SEM) was conducted to further explore the relationships between soil chemical properties and soil fungi under different treatments to some extent. According to this analysis, it is worth mentioning that the soil properties, soil K component, and soil enzyme activity were influenced obviously (*p* < 0.05) by biochar and K fertilizer treatment (Figure 6A). Our SEM analysis found that soil physiochemical properties and K components had weak effects on soil fungi, and soil enzyme activity had a highly significant effect on soil fungi (path coefficient = 0.44) (Figure 6A). Moreover, the total effect of SEM further demonstrated that soil enzyme activity was the most important soil variable for the diversity of fungi, especially that of the C-related enzymes, based on the results of the pot experiment (Figure 6B and Table S6).

## 3. Discussion

### 3.1. Changes in Chemical Properties over Time

The majority of existing research shows that the incorporation of biochar into the soil can significantly improve the soil K level and nutrient status [18,38]. Our pot experiment findings indicated that water-soluble K, available K, and exchangeable K decreased over time, whereas the effects of biochar on soil K content appeared to be increasing at the harvest time (Figure 1). Compared to K0 treatment, the AK, WK, EK, and NEK increased by 125.78%, 124.39%, 126.01%, and 26.63% under CK100 treatment. This phenomenon could be explained by the amount of available K that could be utilized by citrus for a long time. However, there are three main explanations for the long-term promotion effect of biochar on K. First, biochar could promote the activity and quantity of soil microorganisms, including K-dissolving bacteria [39]. Second, a large amount of K absorbed on the surface of biochar can provide nutrients, to some extent, for the growth of plants [40,41,42]. Thus, we found that biochar could significantly increase the K content of leaves [43]. Moreover, the positive role of biochar in improving soil (pH and SOM) weakened over time (Table 1). After 30 months, the pH and SOM were increased by 0.09–0.20 units and 103.47–109.97% (Table 1). This may be due to the structure of biochar being destroyed under long-term acid and rain conditions, which decreased the amendment effect of biochar [31,44]. Additionally, Yao et al. [45] found that the aging that process of biochar provides a certain amount of oxygen-containing functional group of carbonyl and carboxyl into the soil, which might explain why biochar can increase the soil pH constantly. However, according to the results of Table 1, the contents of AP and AN were slightly increased several folds during the cultivation process. This was likely due to fertilizer addition during all cultivation stages.

### 3.2. Associations between Fungal and Soil Properties

Several reports have documented that fungi usually grow at a slower rate and survive under nutrient-poorer conditions by utilizing recalcitrant compounds compared to their prokaryotic counterparts [46]. The study results also show that the alpha diversity of fungi was not influenced under biochar and K fertilizer treatments (Figure S2). This may be because the fungus has strong adaptability to the soil environment, so it is hardly affected by the nature of the soil [47,48]. In addition, fungi, referred to as K-strategy microorganisms, prefer to grow in an environment with limited nutrients. Alpha diversity from a subset of soil samples (e.g., K0, K100, CK0, and CK100) was also impacted by soil properties in our pot experiment (Figure 2). It is known that biochar can also reduce soil bulk density, and improve soil porosity and soil water holding capacity [6,26,49]. Mandal et al. [50] found that biochar can significantly improve the water-holding capacity of sandy soil, but has less effect on clay. Then there is the widely held belief that soil texture is more important to soil fungi [51]. For example, silt or clay content can increase the relative abundances of fungal taxa (*Basidiomycota* and *Eurotiomycetes*). Soil fungi are an important part of the soil ecosystem and are tightly related to the biogeochemical cycle of the soil [52]. Moreover, based on the results of the two groups of comparison, fungal relative abundance was not influenced by biochar and K fertilizer addition (Figure 3). It has been estimated that the soil microbes are mainly related to soil’s physical structure in relation to the long-term effects of biochar and K fertilizer. Zhang et al. [53] found that a single application of biochar was more suitable for yellow–brown soil, while co-application of biochar and K fertilizer was more beneficial to black soil.

In our pot experiment, *p_Ascomycota*, *p_Mortierellomycota*, *p_Basidiomycota,* and *p_Glomeromycota* were the main dominant taxa on the soil environment. Liu et al. [54] also found *p_Ascomycota* and *p_Basidiomycota* are the main microorganisms in the black soil of Northeast China. Previous studies have demonstrated that *p_Ascomycetes* and *p_Basidiomycota* can use more resources and better resist environmental pressure to improve their dominant position in oligotrophic soil [55]. Additionally, compared with other fungi, most of these dominant fungi have higher genomic potential in terms of resource utilization, competition, and environmental resilience. Citrus is a perennial woody plant, and its roots can be infested by a variety of root fungi. Most studies have shown that arbuscular fungi (AM) are some of the important factors in the nutrient metabolism of crops, especially in citrus production [56]. Arbuscular mycorrhiza can reduce the pH of plant rhizosphere, improve the soil microenvironment, increase soil nutrient content, promote crop growth, and enhance stress resistance [57,58]. It is also able to explain why *p_Ascomycetes* and *p_Basidiomycota* and some of their subordinate taxa also exhibit obvious associations with soil properties. Furthermore, among the different taxa of fungi, the relationships between fungi and their subclasses and soil properties are somewhat opposite. For example, the *c_Sordariomycetes* and its members were mainly related to soil properties and nutrient status, and had positive relations for soil pH, AN, AP, and βG. *c_Dothideomycetes* and its members showed the strongest negative association with soil properties from the results of pot experiment (pH, AN, AP, Dehy, βG, and CBH) (Figure 4). There are two main explanations for this phenomenon. For example, recent research has shown that factors such as sampling location and environmental factors, such as soil pH, climate, vegetation type, and soil texture, influence the diversity of microbial communities [59,60]. Furthermore, different taxa of fungi play different roles in the soil environment, and their responses to dissimilar soil characteristics differ greatly. The ecological functions related to nutrition (such as phosphate transporters and nitrogen fixation) and carbohydrate metabolism (such as degradation of complex sugars and polysaccharide synthesis) [61,62]. Interestingly, the results of stepwise multiple regression are also consistent with the previous conclusion. The most affected fungal phylum by the environment was *p_Basidiomycota*, followed by *p_Basidiomycota* and *p_Mortierellomycota* (Figure 4). By using the RDA and SEM approach, it has been demonstrated that there was a close relationship between the abundance of fungal and soil properties (Figure 5 and Figure 6). The function of CBH and βX intricated with soil C degradation and N cycling had significant influences on the phyla and genera of soil fungal community structure (Table S8). Paul et al. [63] found that the change in the soil fungal community was related to the value of total C/N, and the second factor was the soil pH. Moreover, we observed that the soil properties, soil K components, and soil enzyme activity were influenced by biochar and K fertilizer treatment (Figure 6). Interestingly, the SEM also proved that soil fungi were significantly affected (*p* < 0.05) by soil enzyme activity (path coefficient = 0.44) (Figure 6). Together, these analyses suggest that the environmental conditions of soil fungi, such as soil texture, pH, C/N value, light, moisture, temperature, and latitude, have a certain impact on the soil fungus community.

## 4. Materials and Methods

### 4.1. Experimental Materials

In this experiment, the soil was collected at a depth of 0–20 cm from Xianning, Hubei province (114°17′ E, 29°53′ N). According to the soil classification, it is a eutroferric red latosol. In China, the peanut (*Arachis hypogaea L*.) is an important leguminous crop that usually grows in acidic soils [64]. After the soil was naturally air-dried, the soil sample was completely mixed and passed through a 2 mm sieve to remove roots and stones. The biochar material was a peanut shell material (400 °C, 4 h, provided by Shenyang Agricultural University). The peanut shell material was cut into small segments for pyrolysis in the continuous multiple hearth kiln: 20 °C was the initial temperature, 5 L of gas was flowing at a rate of 15 °C per minute, a residence time of 20 min. CO_2_ was the pyrolysis atmosphere. The whole preparation process was carried out under the condition of low O_2_ [51]. The details of the chemical properties of the basic soil and peanut biochar are shown in Table S1. The plants were citrus (*Citrus sinensis Osbeck “Newhall”*) grafted on *Poncirus trifoliata (L.)* (obtained from Ganzhou, Jiangxi, China).

### 4.2. Experimental Design and Management

This experiment was a pot experiment (diameter = 27 cm; height = 30 cm). Every pot had 10 kg of air-dried soil. The research was conducted in 24 pots (three replicates * two biochar levels * four potash fertilizer levels). The detailed fertilization treatments are shown in Table 2. The cultivation experiment was carried out at Huazhong Agricultural University (114°21′ E, 30°28′ N), Wuhan, Hubei province, and citrus plants were sown in pots in April 2018. Before the citrus plants were transplanted, we thoroughly mixed the fertilizer, biochar, and soil. Finally, the method of artificial irrigation and counterweighting was adopted to keep the water holding capacity at about 75%. According to the regularity of the long-term occurrence of pests and diseases, a unified management strategy for pest control efforts was implemented.

### 4.3. Soil Sample and Analyses

Soil samples were collected on the sixth, twelfth, eighteenth, twenty-fourth, and thirty months. The collected soil samples were protected from the interference of the crop rhizosphere effect. The soil sample was about 100 g at every time, collected with a soil drill. The soil sample was divided into 3 parts. One part of each soil sample was stored in liquid nitrogen for soil fungus determination; one part of the soil sample was stored in a refrigerator at 4 °C for soil enzymatic analysis; and the rest of the sample was air-dried and passed through 20 mesh and 100 mesh sieves for determination of chemical properties.

The chemical properties of the soil were determined according to the method described by Bao. [65]. The pH was measured in a 1:2.5 (*w*/*v*) suspension in deionized water with a digital pH meter (FE20/EL20, Shanghai Mettler Toledo Co., Ltd., Shanghai, China). The OM was measured by the K dichromate volumetric method. The available N and P (AN and AP) were measured by the alkaline diffusion method and extracted with 1 mol·L^−1^ NaHCO_3_ and measured by ultraviolet spectrophotometry (TU-1810, Beijing Persee General Instrument Co., Ltd., Beijing, China). The soil K was determined by flame photometer method (AP-1200, Shanghai Precision Instrument Co., Ltd., Shanghai, China), in which acid soluble K (ASK), water-soluble K (WK), available K (AK), and total K (TK) were leached with 1 mol·L^−1^ hot nitric acid, deionized water, 1 mol·L^−1^ NH_4_OAc, and NaOH. The different forms of K were calculated as follows: exchangeable K (EK) = available K – water-soluble K; non-exchangeable K (NEK) = acid-soluble K – available K; structural K (SK) = total K – acid soluble K.

The activities of soil urea and dehydrogenase (Dehy) were determined with a soil kit (Jiangsu Meibiao Biotechnology Co., Ltd., Yancheng, China) [43]. Specifically, 9 mL of neutral phosphate buffer (pH: 6.80) was mixed with 1 g of randomly fresh soil and incubated at 4 °C for 20 min at a speed of 2500 r/min. Then, the supernatant was collected to test enzyme activity with the soil enzyme kit. The other extracellular enzymes (N-acetyl-β-D-glucosaminidase, NAG; β-glucanase, βG; Cellobiohydrolase, CBH; β-Xylosidase, βX; and phosphatase, Phose) were measured with the microplate fluorimetric assay method [66,67,68]. The details of the detection process were as follows: First, approximately 2.0 g of fresh soil was added to a 250 mL plastic bottle containing 200 mL of 50 mM acetate buffer. Then, the soil suspension and substrate were incubated at 25 °C for 4 h, and absorption was detected with a multifunctional microplate reader at 365 and 450 nm (Scientific Fluoroskan Ascent FL, Thermo, Beijing, China), as reported in our previous study [44].

### 4.4. Soil DNA Extraction, PCR, and Sequencing

We used ITS1F (5′-CTTGGTCATTTAGAGGAAGTAA-3′) and ITS2R (5′-GCTGCGTTCTTCATCGATGC-3′) for fungal-community composition and diversity analysis [69]. The amplification system and Illumina Miseq sequencing process were analyzed according to previous related research in our laboratory [32]. The detailed information of system information and the thermal profile of qPCR are shown in Table S2.

All sample sequences were flattened according to the minimum sequence number, the original sequencing sequence used Trimmomatic for quality control, and FLASH was used for sequence splicing [70]. We performed OTU clustering of sequences based on 97% similarity and used UCHIME to eliminate chimeras [71]. Finally, we used the RDP classifier to annotate all microbial species (Unite 7.0 database for fungi) and set the threshold value to 70% [72].

### 4.5. Statistical Analysis

The results are given as mean ± standard deviation, and all data were analyzed with ANOVA. The Duncan test was used to compare the means for variables (*p* < 0.05) (SPSS 25.0). All the data of soil fungi were analyzed on the free online Majorbio Cloud Platform (www.majorbio.com, accessed on 24 December 2020). The alpha diversity index of fungi was analyzed by Student’s t-test to calculate the differences among all treatments. The diversity index, principal component analysis, and network analysis were operated by free online R software (Version 4.2.1) [73].

## 5. Conclusions

Our pot experiment demonstrated that biochar could continuously provide soil K content for two years compared to without biochar addition. Therefore, we argued that the special porous structure of biochar and its nutrient supply are sufficient to explain why it has a visible result on soil K. Moreover, our data indicated the improvements of biochar in soil pH, available nutrients, and organic matter through the results of the pot experiment. We conclude that variations in the effects of biochar should be taken into consideration in the aging of biochar in the long term in acid soil. As an important biological group, soil fungi play an important role in the soil ecosystem. The pot experiment results of biochar illustrate the unique effects on soil properties and fungal communities. First, although there was no significant difference in fungal diversity, there was a significant correlation between fungal diversity and soil characteristics under different treatments. Second, our findings suggest that *p_Ascomycota* and *p_Basidiomycota* were the dominant populations in the soil, and their sub-levels had varying correlations with soil properties. Third, we also proved that soil fungi change with the changes in the soil environment—a dynamic process of constant change—by using the SMR and SEM approach. Thus far, the breadth and depth of research on soil microbial ecology are still very limited. The functional properties of many soil fungi and their roles in communities and ecosystems need to be further studied to provide a stronger basis for agricultural production, disease control, ecological protection, and fungal resource development. Moreover, field experiments should be conducted to further verify our results in our future studies.

## Figures and Tables

**Table 1:** The physicochemical characteristics of soil in different sampling times during all cultivation progress under biochar and K fertilizer treatment.

pH	6 Month	12 Month	18 Month	24 Month	30 Month	SOM	6 Month	12 Month	18 Month	24 Month	30 Month
K0	4.73 ± 0.06 b	4.76 ± 0.73 b	5.07 ± 0.06 a	6.26 ± 0.10 ab	6.75 ± 0.02 bc	K0	17.27 ± 2.44 c	16.50 ± 0.80 bc	11.10 ± 1.28 c	9.79 ± 0.62 c	8.06 ± 0.86 b
K60	4.65 ± 0.04 bc	4.74 ± 0.02 b	5.07 ± 0.07 a	6.15 ± 0.04 abc	6.70 ± 0.01 cd	K60	15.83 ± 1.13 c	15.41 ± 0.70 c	11.33 ± 1.00 c	9.45 ± 0.82 c	7.92 ± 0.82 b
K80	4.56 ± 0.06 c	4.72 ± 0.02 b	5.02 ± 0.12 a	6.13 ± 0.06 bc	6.64 ± 0.04 d	K80	15.07 ± 1.25 c	14.43 ± 1.41 c	11.69 ± 1.39 c	9.29 ± 0.89 c	7.40 ± 0.71 b
K100	4.55 ± 0.03 c	4.70 ± 0.01 b	5.03 ± 0.09 a	5.97 ± 0.23 c	6.53 ± 0.09 e	K100	14.47 ± 0.51 c	10.42 ± 0.63 d	10.73 ± 0.48 c	7.97 ± 0.29 d	7.22 ± 0.96 b
CK0	5.11 ± 0.05 a	5.18 ± 0.29 a	6.00 ± 0.07 b	6.37 ± 0.04 a	6.87 ± 0.04 a	CK0	29.00 ± 4.89 b	20.58 ± 1.57 b	15.42 ± 1.44 b	14.71 ± 0.91 b	15.16 ± 1.47 a
CK60	5.06 ± 0.12 a	5.14 ± 0.03 a	6.00 ± 0.05 b	6.31 ± 0.20 ab	6.79 ± 0.01 b	CK60	32.18 ± 5.56 ab	33.63 ± 3.47 a	18.60 ± 1.79 a	16.81 ± 0.28 a	16.40 ± 0.95 a
CK80	5.07 ± 0.03 a	5.14 ± 0.07 a	5.95 ± 0.09 b	6.31 ± 0.03 ab	6.76 ± 0.04 bc	CK80	35.06 ± 2.27 a	30.93 ± 2.47 a	17.41 ± 1.86 ab	16.55 ± 0.95 a	15.62 ± 0.60 a
CK100	5.02 ± 0.04 a	5.08 ± 0.03 a	5.90 ± 0.12 b	6.22 ± 0.05 ab	6.73 ± 0.02 bc	CK100	34.78 ± 2.88 a	29.81 ± 1.54 a	17.27 ± 2.52 ab	14.31 ± 1.71 b	15.25 ± 0.69 a
AN	6 month	12 month	18 month	24 month	30 month	AP	6 month	12 month	18 month	24 month	30 month
K0	78.17 ± 8.08 a	56.29 ± 5.83 a	68.54 ± 2.53 ab	60.78 ± 3.54 ab	66.50 ± 6.06 ab	K0	5.77 ± 0.66 c	5.91 ± 0.62 c	15.49 ± 2.12 a	12.30 ± 2.73 a	33.01 ± 2.80 abc
K60	77.00 ± 3.50 a	56.88 ± 8.35 a	64.17 ± 2.02 bc	55.24 ± 4.04 bc	63.29 ± 0.51 ab	K60	6.24 ± 1.01 c	6.20 ± 0.54 c	16.37 ± 1.01 a	14.29 ± 1.01 a	29.27 ± 4.49 bcd
K80	78.17 ± 5.35 a	53.67 ± 2.03 a	64.17 ± 4.04 bc	55.24 ± 2.02 bc	61.25 ± 1.75 ab	K80	8.75 ± 0.65 b	7.12 ± 0.77 bc	16.86 ± 2.27 a	15.43 ± 2.18 a	27.49 ± 1.19 cd
K100	77.00 ± 3.50 a	53.67 ± 2.03 a	58.33 ± 2.02 d	53.20 ± 2.32 c	58.92 ± 7.07 b	K100	9.39 ± 1.22 b	7.05 ± 1.17 bc	17.14 ± 3.01 a	14.75 ± 1.44 a	26.12 ± 1.79 d
CK0	80.50 ± 3.50 a	56.00 ± 3.5 a	71.17 ± 5.35 a	66.33 ± 3.03 a	67.38 ± 2.32 a	CK0	9.92 ± 1.10 b	8.06 ± 1.00 ab	17.74 ± 1.85 a	13.67 ± 2.18 a	35.94 ± 3.85 a
CK60	85.17 ± 4.04 a	53.67 ± 2.02 a	65.04 ± 1.82 bc	63.41 ± 5.35 a	64.17 ± 4.04 ab	CK60	10.46 ± 1.46 ab	9.64 ± 1.41 a	18.08 ± 0.72 a	14.55 ± 2.10 a	34.51 ± 4.18 ab
CK80	84.00 ± 3.50 a	52.50 ± 3.50 a	63.88 ± 2.32 bc	55.24 ± 1.01 bc	61.54 ± 2.53 ab	CK80	12.03 ± 0.84 a	9.77 ± 0.85 a	18.02 ± 2.01 a	15.59 ± 1.77 a	31.56 ± 0.79 abcd
CK100	83.13 ± 6.95 a	52.21 ± 3.07 a	60.67 ± 2.02 cd	54.66 ± 3.64 bc	60.38 ± 3.81 ab	CK100	12.40 ± 1.83 a	9.75 ± 0.65 a	18.09 ± 1.83 a	15.55 ± 0.82 a	32.23 ± 3.81 abc

Note: Values show replicate plot means (*n* = 3) and the standard error of the mean (S.E.M.). Colored cells denote heatmap visualization of the differences between treatments with column means. Values followed by a different lowercase letter indicate significant differences according to Duncan’s test (*p* < 0.05).

**Table 2:** Overview of the experimental plan and design.

Treatment		Abbreviation
Biochar treatment	0% K	CK0
	KNO_3_ 0 mg·kg^−1^	
	60% K	CK60
	KNO_3_ 364 mg·kg^−1^	
2%	80% K	CK80
	KNO_3_ 485 mg·kg^−1^	
	100% K	CK100
	KNO_3_ 606 mg·kg^−1^	
Non-biochar treatment	0% K	K0
	KNO_3_ 0 mg·kg^−1^	
	60% K	K60
	KNO_3_ 364 mg·kg^−1^	
0%	80% K	K80
	KNO_3_ 485 mg·kg^−1^	
	100% K	K100
	KNO_3_ 606 mg·kg^−1^	
Other fertilizer management	CO(NH_2_)_2_ 402 mg·kg^−1^, Na_2_HPO_4_·12H_2_O 356.3 mg·kg^−1^, CaCO_3_ 719 mg·kg^−1^, MgSO_4_·7H_2_O 492 mg·kg^−1^	

Note: Since KNO_3_ was used in this experiment, it was necessary to add CO(NH_2_)_2_ while reducing the application of potassium to ensure the same amount of nitrogen was applied in each treatment.

## Data Availability

The availability of data and materials is on the basis of personal request.

## Supplementary Materials

The following supporting information can be downloaded at: https://www.mdpi.com/article/10.3390/plants11243442/s1, Table S1: The physical and chemical properties of the basic soil and peanut biochar; Table S2: The system information and thermal profile of qPCR; Table S3: The differences of on soil physicochemical characteristics during all cultivation progress under biochar and K fertilizer treatment; Table S4: The differences of different forms of soil potassium during all cultivation under biochar and K fertilizer treatment; Table S5: The main effect and interaction effect of different factors; Figure S1: The alpha index of soil fungi between different treatments; Figure S2: The percent of community abundance on phylum level under different treatment (A). Lefse analysis identities microbial biomarkers in each treatment (B); Table S6: The datasheet for environmental factors in RDA on phylum and genus level.
