# Supplementary material for: 30-Month Pot Experiment: Biochar Alters Soil Potassium Forms, Soil Properties and Soil Fungal Diversity and Composition in Acidic Soil of Southern China
Source: Plants (Basel). 2022 Dec 9;11(24):3442. doi: 10.3390/plants11243442 (PMC9783735; doi:10.3390/plants11243442)
Supplement: Supplementary file 1 [file plants-11-03442-s001.zip › plants-2046177-supplementary.pdf]

Table S1The physical and chemical properties of the basic soil and peanut biochar

Parameters	Soil	Parameters	Biochar
pH (H_2O)2.5:1	4.62±0.04	pH (H_2O)2.5:1	8.76
Organic matter (g kg^{-1})	16.45±1.06	Organic matter (g kg^{-1})	555.1
AN (g kg^{-1})	72.33±5.35	TN (g kg^{-1})	18.84
AP (g kg^{-1})	4.66±1.06	TP (g kg^{-1})	2.59
AK (g kg^{-1})	149.33±3.21	TK (g kg^{-1})	8.48
WK (g kg^{-1})	35.00±1.00	Ca (g kg^{-1})	26
NEK (g kg^{-1})	262.67±9.07	Mg (g kg^{-1})	7.7
EK (mg kg^{-1})	114.33±3.21	Mn (mg kg^{-1})	231.1
		Cu (mg kg^{-1})	43.98
		Zn (mg kg^{-1})	62.92

Table S2 The system information and thermal profile of qPCR

System	Volume(μL)	Thermal profile	Processing
5×Fast Pfu Buffer	4	Pre-denaturation	95 °C for 3 min
2.5 m M d NTPs	2	deformation	95 °C for 30 s
Upstream primer (10μM)	0.8	annealing	55 °C for 30 s
Downstream primer (10μM)	0.8	elongation	72 °C for 30 s
Fast Pfu Polymerase	0.4	extension	72 °C for 10 min
DNA template	10ng		
dd H_2O	Add to 20		

Table S3 The differences of on soil physicochemical characteristics during all cultivation progress under biochar and K fertilizer treatment

pH	6month	12month	18month	24month	30month	OM	6month	12month	18month	24month	30month
K0	D	D	C	B	A	K0	A	B	C	CD	D
K60	E	D	C	B	A	K60	A	B	B	C	C
K80	E	D	C	B	A	K80	A	B	B	C	C
K100	D	D	C	B	A	K100	A	B	B	C	C
CK0	D	D	C	B	A	CK0	A	B	C	C	C
CK60	D	D	C	B	A	CK60	A	A	B	B	B
CK80	D	D	C	B	A	CK80	A	A	B	B	B
CK100	D	D	C	B	A	CK100	A	A	B	B	B
**AN**	**6month**	**12month**	**18month**	**24month**	**30month**	**AP**	**6month**	**12month**	**18month**	**24month**	**30month**
K0	A	C	AB	BC	BC	K0	C	C	B	B	A
K60	A	BC	B	BC	C	K60	C	C	B	B	A
K80	A	D	B	CD	BC	K80	C	C	B	B	A
K100	A	B	B	B	B	K100	C	C	B	B	A
CK0	A	C	B	B	B	CK0	CD	D	B	BC	A
CK60	A	C	B	B	B	CK60	CD	C	B	BC	A
CK80	A	C	B	C	B	CK80	C	C	B	B	A
CK100	C	C	B	B	A	CK100	CD	C	B	BC	A

Note: Columns with different letters (A, B … …) indicate significant differences among different sampling time at the 5% probability level. * and ** indicate significant results by F-test at $p < 0.05$ and $p < 0.01$, respectively.

Table S4 The differences of different forms of soil potassium during all cultivation under biochar and K fertilizer treatment

AK	6month	12month	18month	24month	30month	WK	6month	12month	18month	24month	30month
K0	A	A	A	B	C	K0	A	A	A	A	B
K60	A	A	A	AB	B	K60	A	A	A	B	C
K80	A	B	C	C	D	K80	A	A	A	B	B
K100	A	B	C	C	C	K100	A	A	A	B	B
CK0	A	A	A	B	B	CK0	A	A	A	B	B
CK60	A	AB	B	C	C	CK60	A	A	A	B	B
CK80	A	AB	BC	CD	D	CK80	A	A	A	B	B
CK100	A	AB	B	C	D	CK100	A	A	A	B	B
EK	6month	12month	18month	24month	30month	NEK	6month	12month	18month	24month	30month
K0	A	AB	AB	BC	C	K0	B	B	A	A	A
K60	A	A	A	A	A	K60	B	B	A	A	A
K80	A	AB	B	B	B	K80	B	AB	AB	A	A
K100	A	AB	AB	B	B	K100	C	C	B	A	AB
CK0	A	A	A	A	A	CK0	A	A	A	A	A
CK60	A	A	A	A	A	CK60	B	B	AB	A	AB
CK80	A	A	A	A	A	CK80	B	B	AB	A	AB
CK100	A	A	A	A	A	CK100	C	C	BC	A	AB

Note: Columns with different letters (A, B … …) indicate significant differences among different sampling time at the 5% probability level. * and ** indicate significant results by F-test at $P < 0.05$ and $P < 0.01$, respectively.

Table S5 The main effect and interaction effect of different factors.

Factors	pH	SOM	AN	AP	AK	WK	EK	NEK
C	656.53**	959.81**	7.56**	54.64**	1983.09**	579.21**	800.95**	95.63**
F	12.79**	4.43**	11.94**	0.43	525.38**	115.42**	238.13**	19.16**
T	2030.36**	194.11**	146.68**	545.18**	94.96**	197.17**	13.31**	48.53**
C*F	0.443	12.38**	0.327	0.176	23.73**	4.37**	14.17**	0.73
C*T	74.75**	54.19**	2.79*	3.99**	3.93**	31.82**	0.354	2.50*
F*T	0.61	1.11	1.62	3.90**	1.44	1.79	0.81	0.56
C*F*T	0.41	2.75**	0.46	0.28	0.94**	2.73	0.49	0.44

Note: C, F and T indicated biochar, K fertilizer and time factor, respectively; * and ** indicate significant results by F-test at $P < 0.05$ and $P < 0.01$, respectively.

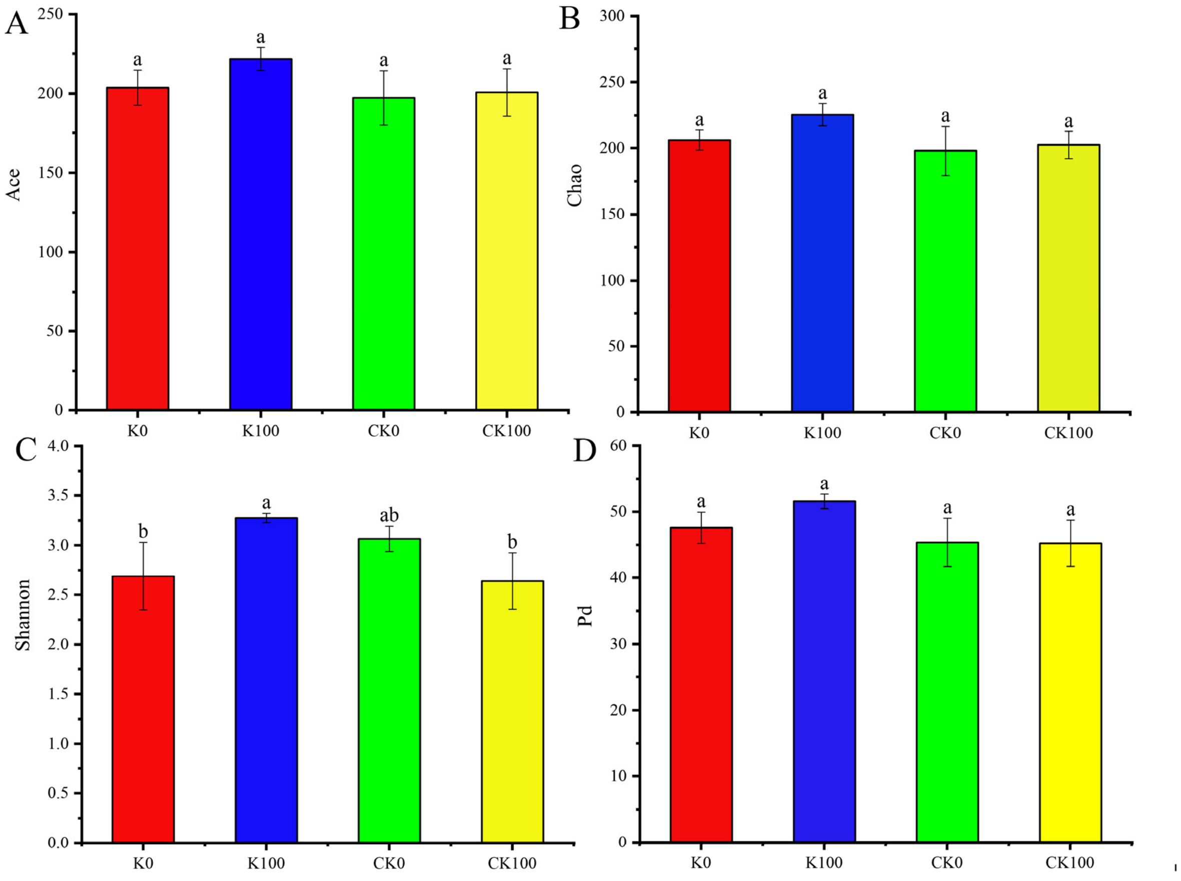

Figure S1 The alpha index of soil fungi between different treatments. Columns with different letters (a, b … …) indicate significant differences among different sampling time at the 5% probability level.

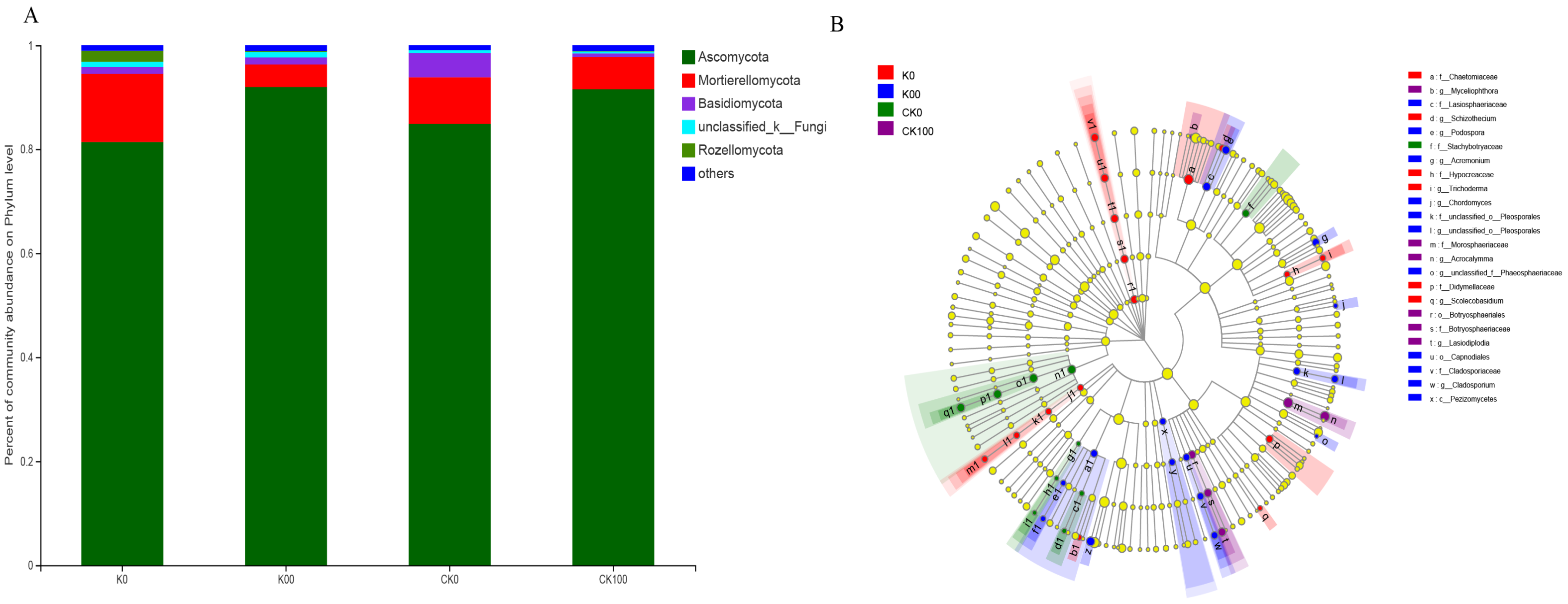

Figure S2 The percent of community abundance on phylum level under different treatment (A). Lefse analysis identities microbial biomarkers in each treatment(B). The yellow node represents that the species is not significantly enriched, and the nodes of other colors represent that the species are enriched in corresponding treatment.

Table S6 The datasheet for environmental factors in RDA on phylum and genus level.

Index	Phylum				Genus			
	RDA1	RDA2	r2	p_values	RDA1	RDA2	r2	p_values
pH	-0.614	-0.790	0.291	0.231	0.649	-0.761	0.009	0.975
AP	-0.692	-0.722	0.154	0.503	0.880	-0.475	0.198	0.377
AN	-0.951	-0.311	0.048	0.790	-0.799	-0.602	0.225	0.356
SOM	0.098	-0.995	0.117	0.637	-0.959	0.283	0.495	**0.045**
WK	0.938	0.347	0.223	0.369	-0.996	-0.085	0.380	0.107
NEK	0.804	-0.595	0.121	0.609	-0.997	-0.075	0.696	**0.003**
EK	0.996	-0.087	0.508	**0.038**	-0.967	-0.254	0.187	0.388
SK	0.538	-0.843	0.088	0.693	0.979	-0.206	0.194	0.381
Dehy	-0.786	0.619	0.103	0.525	-0.158	0.988	0.070	0.718
Urea	0.798	0.602	0.375	0.142	-0.983	0.183	0.261	0.273
NAG	0.303	0.953	0.154	0.522	-0.947	0.321	0.331	0.190
βG	-0.710	0.704	0.599	**0.008**	0.603	0.798	0.416	0.101
CBH	-0.315	0.949	0.678	**0.006**	-0.071	0.998	0.566	**0.041**
BX	-0.305	0.953	0.752	**0.004**	-0.175	0.985	0.563	**0.035**
Phos	-0.954	-0.299	0.408	0.085	0.987	-0.161	0.566	**0.025**

Note: pH, AN, AP, OM, WK, NEK, EK, SK, Urea, Dehy, NAG, βG, CBH, βX, Phos represent pH, alkali nitrogen, available phosphorus, organic matter, Water-soluable K, Non- Exchangeable K, Exchangeable K, Structural K, Urea, Dehydrogenase, N-acetyl-β-D-glucosaminidase; β-glucanase; Cellobiohydrolase; β-Xylosidase and acid phosphatase.
